# Selective Etching of Multi-Stacked Epitaxial Si_1-x_Ge_x_ on Si Using CF_4_/N_2_ and CF_4_/O_2_ Plasma Chemistries for 3D Device Applications

**DOI:** 10.3390/ma18184417

**Published:** 2025-09-22

**Authors:** Jihye Kim, Joosung Kang, Dongmin Yoon, U-in Chung, Dae-Hong Ko

**Affiliations:** 1 Department of Materials Science and Engineering, College of Engineering, Yonsei University, 50, Yonsei-ro, Seodaemun-gu, Seoul 03722, Republic of Korea; paw233@yonsei.ac.kr (J.K.);; 2 BIO-IT Micro Fab Center, Yonsei University, Seoul 03722, Republic of Korea

**Keywords:** Si, SiGe, dry etching, selective etching, CF_4_, N_2_, F radical

## Abstract

The SiGe/Si multilayer is a critical component for fabricating stacked Si channel structures for next-generation three-dimensional (3D) logic and 3D dynamic random-access memory (3D-DRAM) devices. Achieving these structures necessitates highly selective SiGe etching. Herein, CF_4_/O_2_ and CF_4_/N_2_ gas chemistries were employed to elucidate and enhance the selective etching mechanism. To clarify the contribution of radicals to the etching process, a nonconducting plate (roof) was placed just above the samples in the plasma chamber to block ion bombardment on the sample surface. The CF_4_/N_2_ gas chemistries demonstrated superior etch selectivity and profile performance compared with the CF_4_/O_2_ gas chemistries. When etching was performed using CF_4_/O_2_ chemistry, the SiGe etch rate decreased compared to that obtained with pure CF_4_. This reduction is attributed to surface oxidation induced by O_2_, which suppressed the etch rate. By minimizing the ion collisions on the samples with the roof, higher selectivity, and a better etch profile were obtained even in the CF_4_/N_2_ gas chemistries. Under high-N_2_-flow conditions, X-ray photoelectron spectroscopy revealed increased surface concentrations of GeF*_x_* species and confirmed the presence of Si–N bond, which inhibited Si etching by fluorine radicals. A higher concentration of GeF*_x_* species enhanced SiGe layer etching, whereas Si–N bonds inhibited etching on the Si layer. The passivation of the Si layer and the promotion of adhesion of etching species such as F on the SiGe layer are crucial for highly selective etching in addition to etching with pure radicals. This study provides valuable insights into the mechanisms governing selective SiGe etching, offering practical guidance for optimizing fabrication processes of next-generation Si channel and complementary field-effect transistor (CFET) devices.

## 1. Introduction

Because of the scaling down of planar logic devices and their ensuing physical limitations, a new three-dimensional (3D) architecture for devices based on stacked Si channels has emerged. When fabricating structures with this architecture, it is essential to alternate between the epitaxial growth of Si and SiGe, followed by the selective etching of SiGe.

Recently, methods, such as the wet etching method, thermal etching with HCl, and dry etching with plasma have been employed for selective etching of SiGe. Among these, wet etching showed a selectivity of over 150:1 in a Si_0.76_Ge_0.24_ multilayer (ML) structure with a 40 nm layer [1]. However, such high-selectivity wet etching has several limitations. First, if the surface oxidation rate is insufficient, SiGe may remain in the etch tunnel [2]. This can hinder the removal of SiGe, potentially reducing selectivity. Second, the drying process after wet etching can lead to pattern collapse [3,4]. For these reasons, dry etching methods for SiGe removal have gained increasing attention.

HCl thermal etching is one approach used for the selective removal of SiGe [5]. However, it requires high temperatures above 600 °C. This high-temperature process can lead to the undesired diffusion of dopants. Therefore, it is considered unsuitable for Si-based semiconductor processes.

Remote plasma can minimize ion bombardment, thereby reducing unwanted substrate modifications. In inductively coupled plasma reactive ion etching (ICP-RIE), the plasma is generated near the substrate, leading to increased dissociation and allowing more reactive species to be available for the etching process. This results in high plasma density, which contributes to both high reactivity and etch rate. Given these advantages, ICP-RIE is proposed as a promising method for selective SiGe etching, as it is a production-effective process [6]. However, ion bombardment in the ICP chamber may cause structural collapse in 3D structures. Thus, etching with radicals alone (without ion bombardment) is highly recommended.

One selective etching mechanism is the selective oxidation of Ge in the SiGe layer. For example, a CF_4_/O_2_/N_2_ gas chemistry allows for the etching of Si compared with SiGe, as the oxidation of germanium (Ge) occurs more readily, leading to the passivation of the SiGe surface while Si is etched away. This indicates that O_2_ gas should be fed to achieve a high selectivity of SiGe over Si [7,8]. However, studies on the role of N_2_ and the corresponding SiGe etching mechanism are limited. In this work, we investigated the effects and mechanisms of the addition of O_2_ and N_2_ additive gases to CF_4_ etching gas on Si/SiGe multilayer etching and the effects of radicals and ions on selectivity.

In addition to the technological importance of selective Si/SiGe etching, the environmental implications of fluorocarbon gases should also be considered. CF_4_ and SF_6_ are recognized as potent greenhouse gases with high global warming potentials (GWPs). In this study, we tried to minimize the environmental impact by carefully limiting gas leakage outside the chamber and employing a scrubber system. Furthermore, although their effectiveness may differ, the possible use of alternative gases such as NF_3_ should also be considered in light of environmental concerns.

## 2. Experimental

A SiGe/Si ML structure was fabricated through epitaxial growth, alternating between Si_0.75_Ge_0.25_ layers with thicknesses of 50, 30, and 10 nm and Si layers with a thickness of 40 nm. This SiGe/Si ML structure was grown using ultrahigh vacuum chemical vapor deposition (UHV-CVD, Jusung Engineering Co., Ltd., Gwangju, Kyeonggi, Republic of Korea) at 550 °C. Disilane (Si_2_H_6_, SK Materials, Yeongju, Republic of Korea) and germane (GeH_4_, SK Materials, Yeongju, Republic of Korea) served as precursors for the Si and SiGe layers, respectively. The SiGe layers were deposited under gas flow conditions of 20-standard cubic centimeters per minute (sccm) for Si_2_H_6_ and 50 sccm for GeH_4_, whereas the Si layers were grown with a Si_2_H_6_ flow rate of 20 sccm.

The fabricated ML was patterned through spin coating using GXR-601(MERCK, Darmstadt, Germany) under sequential conditions: 500 rpm for 10 s, 2000 rpm for 30 s, and 500 rpm for 5 s. The spin-coated ML was prebaked at 95 °C for 90 s and then exposed using a 5-μm line-pattern mask. After exposure, the sample was hard-baked at 110 °C for 90 s, developed for 60 s, rinsed with deionized water for 30 s, and dried with nitrogen.

Trenches on the line-patterned ML were formed using reactive ion etching with SF_6_, CHF_3_, N_2_, O_2_, and Ar gases (Samhung Special Gas, Yeosu, Republic of Korea). After trench formation, the SiGe/Si ML sidewalls were exposed. The SiGe layers were selectively etched using ICP-RIE (ICP-RIE SYSTEM, LAT Co., Ltd., Suwon, Kyeonggi, Republic of Korea) with CF_4_ and additive gases such as N_2_, O_2_, and Cl_2_(Samhung Special Gas, Yeosu, Republic of Korea). A substrate bias power of 0 W was maintained to enhance selectivity during etching. The substrate bias power controls the ion energy incident on the wafer. A setting of 0 W indicates that no additional RF power was applied to the substrate, thereby minimizing ion bombardment and emphasizing chemical etching. In this study, the ICP source power was kept constant at 100 W. The roof, used to block ion bombardment, was made of Teflon with a thickness of approximately 2 mm. By employing the roof structure, the effect of ion bombardment can be mitigated and the ion flux reduced, while radicals are still able to diffuse through the gap and reach the substrate. As a result, the selectivity can be enhanced. Also, the roof was fabricated from Teflon, which exhibits excellent etch resistance. Because it is scarcely etched, the generation of etch by-products is minimal and thus not expected to influence the substrate etching.

To examine the effects of the process conditions, etching experiments were conducted on SiGe and Si blanket layers and on the SiGe/Si ML. The CF_4_/(CF_4_ + N_2_) gas ratio was varied from 16.7% to 100%, the working pressure was set to 10, 30, and 50 mTorr (1 mTorr ≈ 0.13 Pa), and the total gas flow rate was adjusted between 6 and 60 sccm.

Microstructural analysis of the etched ML was performed using field-emission scanning electron microscopy (SEM, JSM-7001F, JEOL, Tokyo, Japan) with an accelerating voltage of 15 kV. To investigate the etched surfaces of SiGe and Si, X-ray photoelectron spectroscopy (XPS) analysis was conducted using a K-alpha system (Thermo VG, Waltham, MA, USA) equipped with an Al Kα X-ray source. The X-ray power was set to 12 kV and 3 mA, and the sampling area had a diameter of 400 μm.

## 3. Results and Discussion

### 3.1. SiGe Selective Etching Using Various Additive Gases

**Figure 1:**
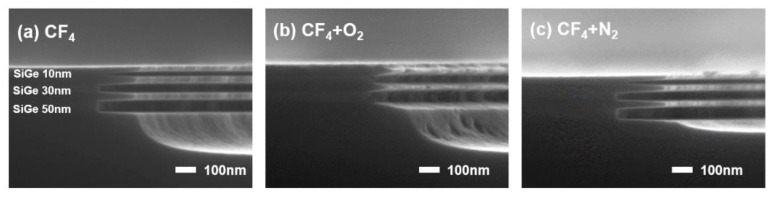
SEM image of the etched SiGe/Si ML with various additive gases.

Figure 1 demonstrates the results of the selective etching of Si_0.75_Ge_0.25_/Si ML using CF_4_ combined with various additive gases in an ICP system, including the etching results with pure CF_4_. Selective etching of Si_0.75_Ge_0.25_ layers with thicknesses of 50, 30, and 10 nm was achieved while keeping the Si thickness at 40 nm. Figure 1 shows that the lateral etch depth decreased as the Si_0.75_Ge_0.25_ layer thickness decreased. Table 1 summarizes the lateral etch depths for each case, showing a reduction in the lateral etch depth as the Si_0.75_Ge_0.25_ layer thickness decreased. This trend could be attributed to the microloading effect [9], which led to a decrease in the etch rates due to the limited diffusion of the by-products and reactant gases in the fine patterns. For the 10 nm thick SiGe layer, the tunnel formed during etching was too narrow for the by-products to escape efficiently, decreasing the lateral etch depth from 183 to 145 nm. If the SiGe layer becomes thinner than 10 nm, the tunnel is expected to narrow further. Consequently, the supply of etchant F radicals and the removal of etching by-products are hindered, leading to a further reduction in the etch rate. A similar effect is also observed in wet etching [1].

Figure 1b shows the results of adding 5 sccm of O_2_ to 30 sccm of CF_4_. Compared with etching in pure CF_4_, the SiGe-to-Si etch selectivity deteriorated. The top layers of both Si and Si_0.75_Ge_0.25_ were almost completely removed. This degradation could be attributed to the introduction of O_2_, leading to the formation of reactive O radicals and promoting the oxidation of the SiGe surface.

Figure 1c depicts the etching outcome with the addition of 5 sccm of N_2_ to 30 sccm of CF_4_. The lateral etch depths for Si_0.75_Ge_0.25_ layers of 50, 30, and 10 nm were deeper than those achieved with CF_4_ alone. From Figure 1, pure CF_4_ or CF_4_ with N_2_ as an additive appears to be a promising gas chemistry for the selective etching of SiGe.

### 3.2. Etch Properties of CF_4_/O_2_ and CF_4_/N_2_ Gas Chemistry Under Various Process Conditions

**Figure 2:**
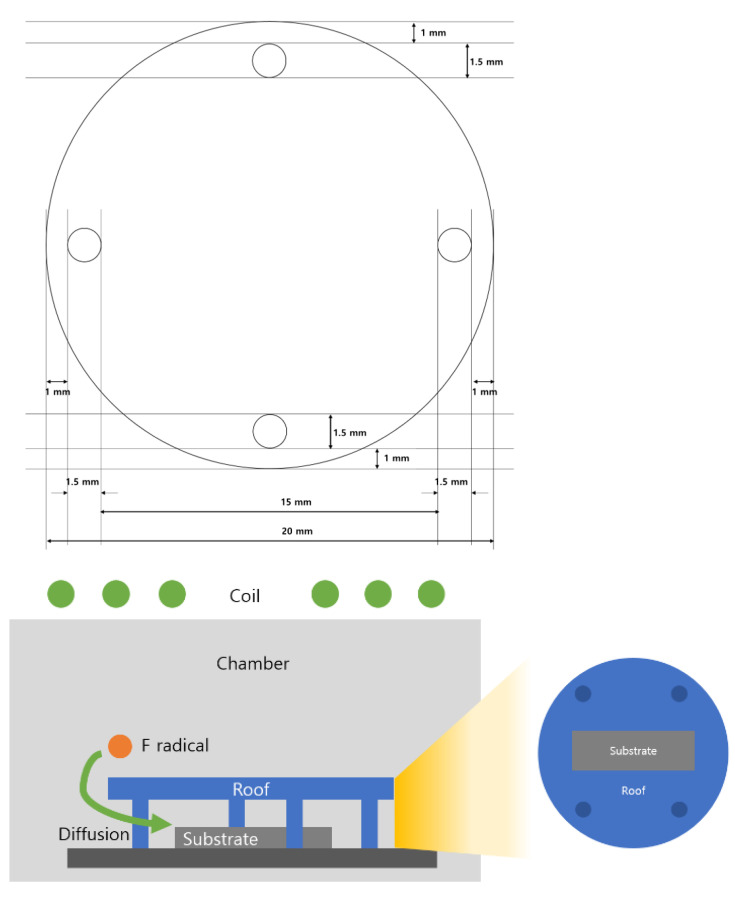
Schematic illustration of the roof structure.

**Figure 3:**
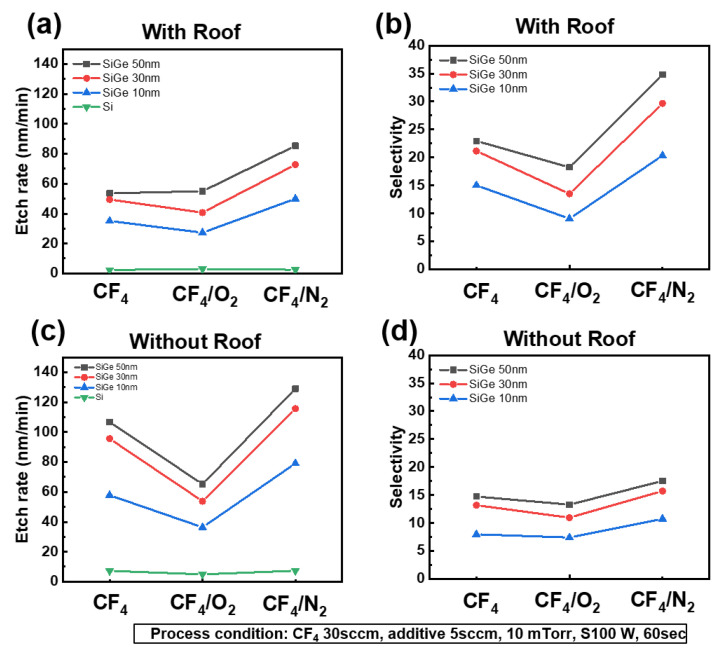
Etch rate (**a**,**c**) and SiGe/Si selectivity (**b**,**d**) under CF_4_ plasma with 5 sccm of O_2_ or N_2_ additive gas, measured for SiGe layers of 10, 30, and 50 nm thickness, with and without a roof structure.

To suppress the self-bias effect, we designed a roof plate. After fabricating the structure shown in Figure 2, we evaluated the etch selectivity with and without the roof. Fluorocarbon species were generated from CF_4_ gas because of the presence of carbon. From a plasma perspective, this phenomenon is attributed to the self-bias effect and occurs even when the substrate bias power is set to 0 W. Figure 3 presents the etch rate and SiGe/Si selectivity under pure CF_4_, CF_4_/O_2_, and CF_4_/N_2_ plasma conditions, with and without the roof. As shown in Figure 3a,c, the addition of O_2_ promoted the formation of a SiO*_x_*F*_y_* passivation layer through surface oxidation, which suppressed the etching reaction and consequently reduced the etch rate [10,11]. In contrast, the addition of N_2_ enhanced CF_4_ dissociation, thereby increasing the etch rate with and without the roof compared with pure CF_4_. This behavior is also evident in Figure 3b,d, where N_2_ addition led to superior selectivity relative to O_2_ addition, indicating that N_2_ is a promising additive for achieving high-selectivity SiGe etching.

A comparison of Figure 3a,c shows that the overall etch rate was higher in the absence of the roof structure. However, without the roof, the enhanced ion bombardment also increased the Si etch rate, reducing the selectivity to approximately 15:1, as illustrated in Figure 3d. In contrast, with the roof, as shown in Figure 3b, the selectivity improved to as high as 35:1 with N_2_ addition, suggesting that ion bombardment accelerated the etching of both SiGe and Si, thereby degrading the selectivity. Therefore, suppressing ion bombardment is essential for achieving high SiGe etch selectivity. For all plasma chemistries and roof conditions, thicker SiGe layers (e.g., 50 nm) exhibited consistently higher etch rates than thinner layers (e.g., 10 nm), potentially because of loading effects. The data presented in Figure 3 emphasize the critical influence of both gas chemistry and ion bombardment modulation on the optimization of selective SiGe etching. Moreover, there were different selective etching mechanisms between the CF_4_/O_2_ and CF_4_/N_2_ gas chemistries.

**Figure 4:**
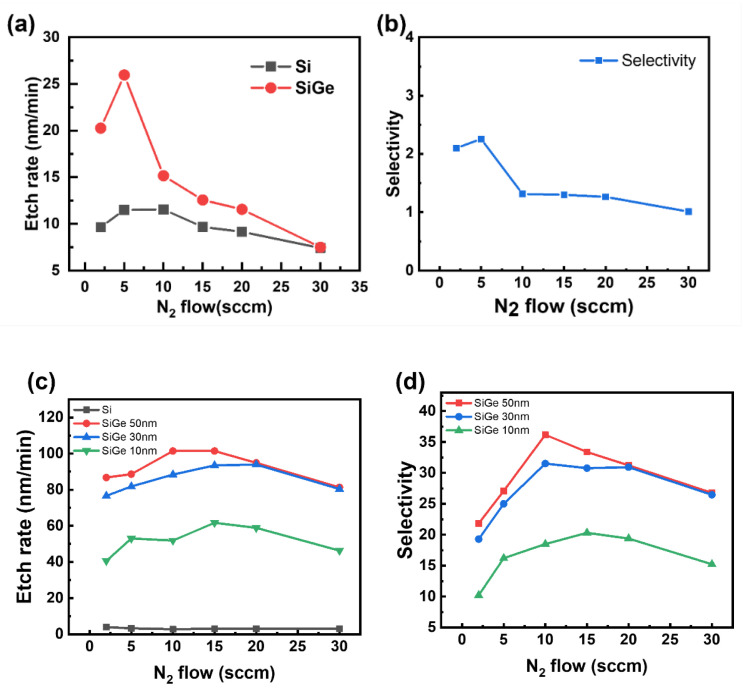
Etch rate (**a**,**c**) and SiGe/Si selectivity (**b**,**d**) under varying N_2_ flows in SiGe/Si multilayers (ML). Etch rate and SiGe/Si selectivity as a function of N_2_ flow rate for (**a**,**b**) blanket films with a roof and (**c**,**d**) ML stacks with varying SiGe thicknesses (10, 30, and 50 nm) with a roof.

Figure 4 presents the etching results as the N_2_ flow rate increased while maintaining a constant partial pressure of CF_4_, with the roof plate placed above the sample. Figure 4a,b show the blanket etching results, while Figure 4c,d present the ML etching outcomes. In Figure 4a, the etch rate reached a maximum of 26 nm/min at an N_2_ flow rate of 5 sccm and then gradually declined with further increases in N_2_. Similarly, Figure 4b shows that the selectivity peaked at 5 sccm and decreased at higher flow rates. A comparable trend appeared in the ML etching results: Figure 4d shows that for all three SiGe thicknesses, the selectivity increased up to 10 sccm and then declined.

The increase in selectivity with moderate N_2_ addition results from two main mechanisms. First, N_2_ enhances CF_4_ dissociation by facilitating the reaction CF_2_ + N→CNF + F, which increases the concentration of reactive F radicals. Second, N_2_ promotes nitrogen passivation (N passivation), which selectively passivates the Si surface, enabling the preferential etching of SiGe. However, excessive N_2_ may lower the electron density in the plasma, reducing the frequency of particle collisions and thereby suppressing the formation of F radicals [12]. The lower electron density suppresses CF_4_ dissociation and subsequently decreases F radical formation. Because the dissociation of fluorocarbon gases depends largely on electron-impact reactions, a decrease in the number of energetic electrons reduces the number of collisions that are energetic enough to break molecular bonds. Consequently, the concentration of the reactive fluorine species in the plasma decreases. Overall, Figure 4 demonstrates that the N_2_ flow rate significantly affects both the etch rate and selectivity, highlighting the importance of optimizing N_2_ flow conditions to achieve effective selective etching.

**Figure 5:**
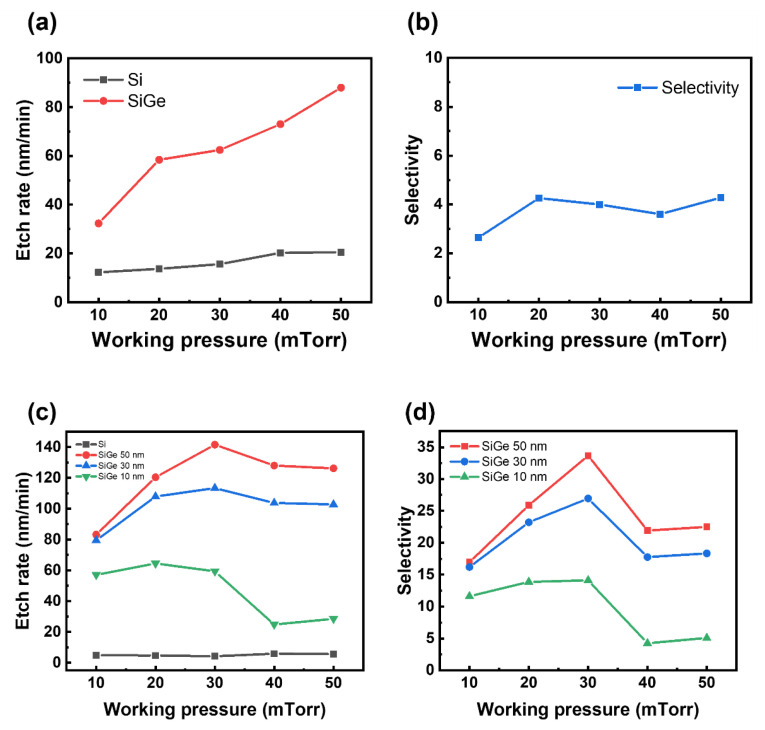
Etch rate and SiGe/Si selectivity as a function of the working pressure. (**a**,**b**) Results from the blanket Si and SiGe films with a roof. (**c**,**d**) Results from the SiGe/Si ML structures with different SiGe thicknesses (10, 30, and 50 nm) with a roof.

Figure 5 shows the etch rate and etch selectivity as functions of the working pressure, keeping the partial pressure of CF_4_ constant and the roof plate fixed, for both the blanket samples and the SiGe/Si ML structures. Figure 5a shows that the etch rates of both SiGe and Si increased with increasing working pressure. This could be attributed to the enhanced supply of fluorine radicals due to the increased collision frequency. Figure 5b presents the selectivity for the blanket samples, which remained relatively unchanged with varying working pressure because the etch rate of Si also increased along with that of SiGe as the pressure increased.

In Figure 5c, the etch rates of the 50 and 30 nm SiGe films increased with the working pressure, reaching peak values of 142 and 113 nm/min, respectively, at 30 mTorr. However, beyond this pressure, the etch rates began to decline, exhibiting a different behavior from those of the blanket wafers. For the 10 nm SiGe film, a significant reduction in the etch rate was observed at pressures exceeding 30 mTorr.

Within the 10–30 mTorr range, increasing the pressure shortens the mean free path (MFP), leading to more frequent collisions and enhanced gas dissociation. This facilitates the generation of fluorine radicals, thereby increasing the etch rate. Additionally, a rise in the working pressure increases the radical-to-ion ratio, contributing to improved etch selectivity [13]. Among the dissociation mechanisms of CF_4_, the primary reaction (CF_4_ + e→CF_3_ + F) that produces F radicals has a relatively low threshold energy of ~5.6 eV, whereas the ionization process (CF_4_ + e→CF_3_^+^ + F + 2e) requires a much higher energy of ~15.9 eV. As the working pressure increases, the average electron energy decreases, making ionization less probable under these conditions [14]. Above 30 mTorr, excessive collisions may cause electrons in the plasma to lose energy, resulting in a lower electron temperature [15]. This, in turn, may suppress the dissociation of process gases and lead to a decreased etch rate. At pressures above a certain threshold, the recombination of reactive species becomes pronounced, which decreases the flux of radicals reaching the substrate [16]. Consequently, such elevated pressures are unsuitable for the etching process. Based on this consideration, 30 mTorr, where the maximum selectivity is achieved, as confirmed in Figure 5d, was selected as the optimal process condition.

In ML structures, unlike in blanket films, structural effects become more pronounced at higher pressures. As the MFP decreases, reactive species struggle to reach sidewall surfaces [16]. This diffusion limitation leads to a reduced etch rate and selectivity when the pressure exceeds a certain threshold.

**Figure 6:**
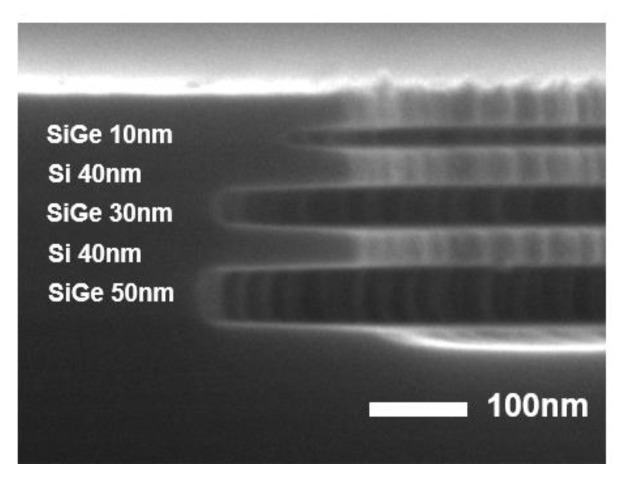
Etched Si_0.75_Ge_0.25_/Si ML under the following conditions: 20 sccm CF_4_, 10 sccm N_2_, and 30 mTorr for 60 s with a roof structure.

Figure 6 presents experimental results comparing the initial and optimized etching conditions for Si_0.75_Ge_0.25_/Si ML structures using ICP-RIE with a roof. The figure includes cross-sectional SEM images along with quantitative data on etch rates and selectivity. The optimized conditions significantly improved the etch rate of the Si_0.75_Ge_0.25_ layer while minimizing that of the Si layer. Moreover, the SEM images illustrate that the ML structure exhibits high etch selectivity with minimal sidewall roughness.

The improved selectivity was due to better control of the etching process, achieved through plasma modulation and surface passivation effects by adding N_2_ gas. By adding an appropriate amount of N_2_ gas to the CF_4_ plasma, the dissociation of CF_4_ was enhanced, which increased the concentration of F radicals (Table 2), leading to a higher SiGe etch rate. In addition, the etch selectivity was significantly improved, reaching up to 37:1. This enhancement is attributed to N passivation, where dissociated N radicals adsorb onto the Si surface and form stable Si–N bonds. These bonds effectively suppress the reaction between the F radicals and the Si surface, thereby protecting Si from etching. As a result, under the optimized process conditions, SiGe was etched, whereas the Si layer was protected by surface passivation. Figure 6 shows the results obtained under these conditions. The optimized pressure conditions suppressed GeF_4_ redeposition and promoted the efficient removal of volatile by-products. In addition, controlled pressure application prevented excessive polymer formation, keeping the etching process governed by surface reactions rather than diffusion-limited transport. The optimized ICP-RIE conditions with the roof structure successfully enhanced the selectivity and etch uniformity in Si_0.75_Ge_0.25_/Si ML structures. These findings highlight that the precise tuning of process parameters significantly enhances etch selectivity, enabling greater precision in semiconductor processing.

Additionally, the general trends of the effects of N_2_ flow and working pressure are not expected to change significantly with Ge concentration. However, since the optimization was performed for Ge 25%, the etch rates and optimal process conditions for other Ge concentrations may differ.

### 3.3. Selective Etching Mechanism According to an XPS Analysis of the Etched Surfaces of SiGe and Si

**Figure 7:**
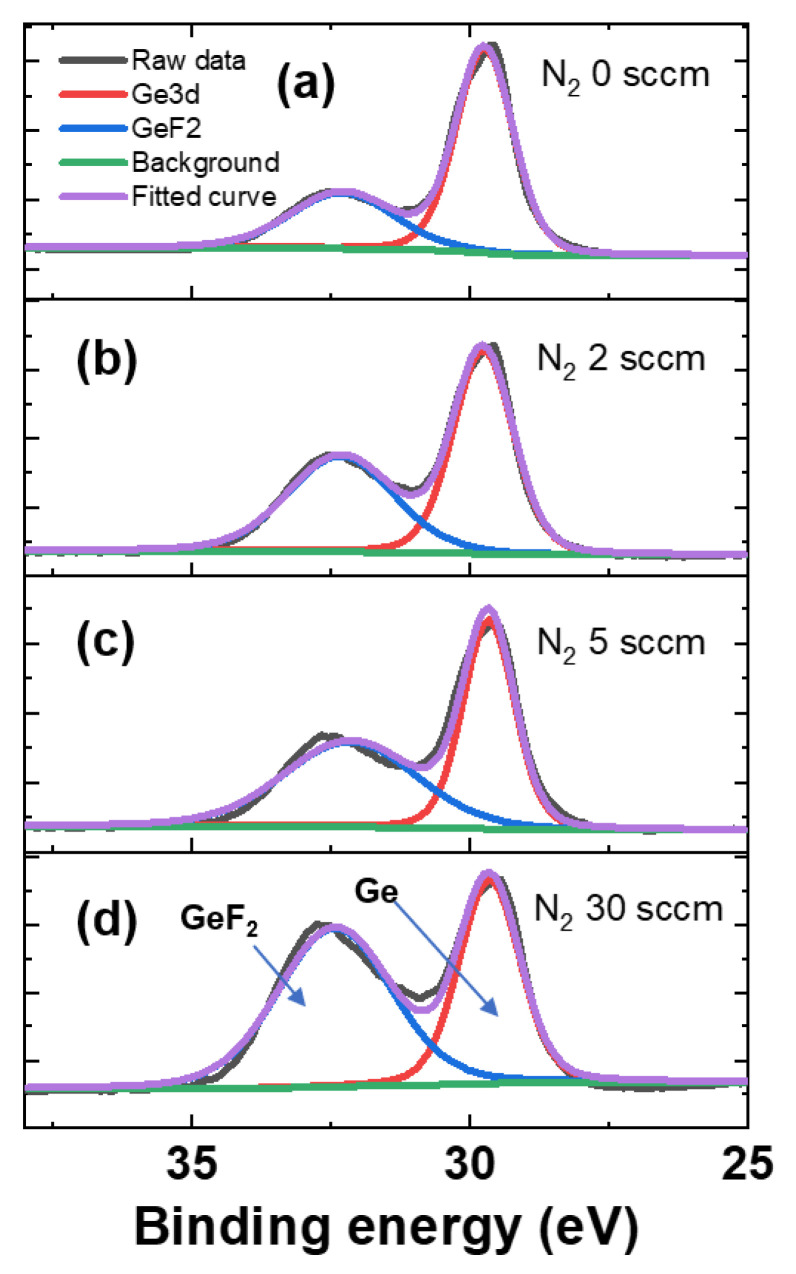
X-ray photoelectron spectroscopy (XPS) Ge 3d narrow-scan spectra for SiGe surfaces etched under different plasma conditions: (**a**) CF_4_ only, (**b**) CF_4_ gas + 2 sccm N_2_, (**c**) CF_4_ + 5 sccm N_2_, and (**d**) CF_4_ + 30 sccm N_2_.

To further investigate the selective etching mechanism, XPS analysis was performed on the etched SiGe surfaces. Figure 7 presents the Ge 3d narrow-scan spectra of the SiGe samples etched under varying N_2_ flow rates while maintaining a fixed CF_4_ partial pressure. Each spectrum revealed two main components: a Ge peak corresponding to elemental Ge (29.7 eV) and a Ge–F peak (32.3 eV) attributed to Ge–F_2_ bonding [17,18]. As shown in Figure 7, GeF_2_ peaks consistently appeared across all conditions, indicating the fluorination of the Ge surface by fluorine radicals generated from CF_4_ dissociation. These F radicals reacted with the dangling bonds of the Ge atoms exposed at the surface, forming Ge–F_2_ bonds, typically observed around 32.5 eV [19].

Quantitative analysis (Table 3) confirms that the fraction of Ge–F_2_ bonding increases with higher N_2_ flow rates. When the N_2_ flow increases up to a certain level, the rise in F radical concentration dominates, leading to a higher etch rate. An increase in N_2_ promotes CF_4_ dissociation, thereby increasing the concentration of F radicals. However, beyond that level, the effect of increased Ge–F_2_ formation on the surface becomes stronger, causing the etch rate to decrease. At high N_2_ flow rates, more GeF_2_ forms on the surface, lowering the etch rate. This is because the remaining fluorine blocks new F radicals from reaching the material, slowing down the etching process [20]. In addition, GeF_2_ has a very high boiling point of approximately 130 °C, making its removal difficult. This inhibitory effect becomes more pronounced with increased N_2_ flow, as the Ge–F_2_ fraction increases from 57.09% at 0 sccm to 79.68% at 30 sccm. These results clearly demonstrate that the GeF_2_ peak intensity is significantly enhanced at 30 sccm, suggesting a strong surface fluorination effect under excessive N_2_ flow conditions.

**Figure 8:**
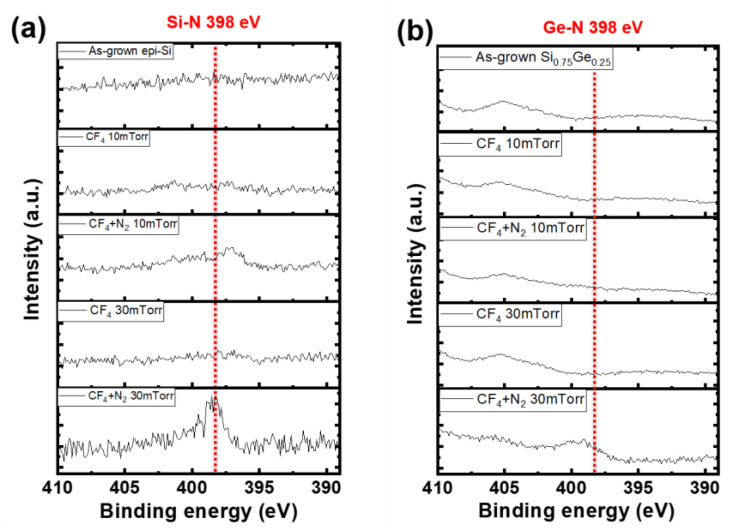
N 1s spectra of (**a**) Si and (**b**) SiGe surfaces obtained under different process pressures with and without N_2_ gas.

Figure 8 shows N 1s XPS spectra under varying working pressures with or without N_2_ addition. As shown in Figure 8b, on the SiGe surface, no distinct N-related peak was observed, or it appeared very broad. Therefore, N-passivation was selectively observed on the Si surface under the 30 mTorr condition. As a result, the formation of Si–N bonds suppressed etching on the Si surface, thereby enhancing the etching selectivity over the SiGe layer. Si–N bonds typically appear at ~398 eV in the N 1s region [21,22]. Herein, a clear Si–N peak appeared under CF_4_ (20 sccm) + N_2_ (10 sccm) plasma at 30 mTorr, confirming that N passivation occurred on the Si surface under these conditions.

Indeed, as shown in Figure 4, increasing the pressure from 10 to 30 mTorr significantly enhanced the selectivity from 17:1 to 34:1 in the ML. These results suggest that both N_2_ addition and process pressure play key roles in enabling nitrogen passivation. Notably, the Si–N peaks did not appear under other conditions, where a broad feature was observed without any sharp peak.

Si–N bonds are inherently more stable and chemically favorable than Ge–N bonds. The dissociation energy of Si–N bonds is approximately 355 kJ/mol, indicating a very strong, readily forming covalent bond [23]. In contrast, Ge–N bonds have a lower dissociation energy (~200 kJ/mol) and are energetically less favorable. Furthermore, considering electronegativity differences (Si, 1.90; Ge, 2.01; and N, 3.04), Si–N bonds exhibit greater ionic character, contributing to their higher stability. Accordingly, nitrogen bonding preferentially occurs on Si rather than Ge, resulting in selective Si surface passivation. Compared with the dangling bond state, the Si–N bonded surface is less reactive with fluorine radicals, leading to improved etch selectivity.

### 3.4. SiGe Etching Mechanism Based on CF_4_/N_2_ Gas Chemistry

**Figure 9:**
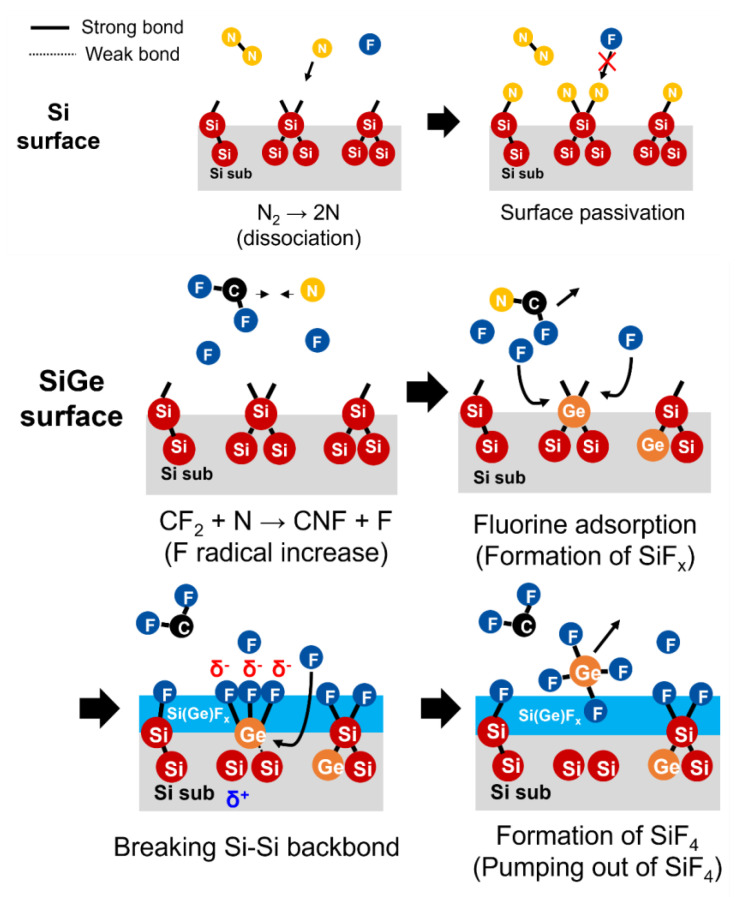
Schematic of the Si/SiGe etching mechanism using CF_4_/N_2_ gas.

Figure 9 illustrates the Si/SiGe etching mechanism using CF_4_/N_2_ gas. On the Si surface, dissociated N_2_ gas produces atomic nitrogen, which bonds with dangling bonds to form Si–N bonds. This N passivation suppresses the adsorption and reaction of fluorine atoms, thereby inhibiting etching—a key factor contributing to improved etch selectivity. In contrast, nitridation is less likely to occur on the Ge surface because of its higher activation energy for nitridation [24].

In a CF_4_/N_2_ mixed plasma, the CF_2_ + N→CNF + F reaction generates additional fluorine radicals, increasing the overall concentration of free fluorine radicals. This enhances fluorine-based reactivity by promoting the adsorption of fluorine radicals onto the dangling bonds on the SiGe surface, resulting in the formation of Si(Ge)F*_x_* species. When multiple F radicals bind to Si or Ge atoms, the electrostatic state of Si or Ge shifts to delta-plus (δ⁺), leading to a charge imbalance. This weakens the Si–Si or Si–Ge backbonds, promoting bond breakage—a critical step in the etching process [25]. As the weakened Si–Si backbonds are attacked by incoming F radicals, the number of Si–F bonds increases, leading to the formation of SiF_4_ from intermediate SiF*_x_* species. Because SiF_4_ has a low boiling point (−86 °C), it becomes a volatile compound that readily desorbs from the surface and is pumped out of the chamber, thereby completing the etching process.

At this stage, the bond energies are 3.25 eV for Si–Si, 3.12 eV for Si–Ge, and 2.84 eV for Ge–Ge. These indicate that the presence of Ge atoms lowers the bond strength [26]. Consequently, the Ge-containing bonds are etched more readily, resulting in a faster etch rate for Ge than for Si.

Finally, the Si(Ge)F*_x_* species react with additional fluorine radicals to form volatile Si(Ge)F_4_, which is then removed from the surface by the plasma environment and pumped out of the chamber, completing the etching process.

## 4. Conclusions

This work investigated the etching mechanism of SiGe using CF_4_/N_2_ gas chemistry in an ICP-RIE system. In this system, ion bombardment is inherently present and can degrade etch selectivity. Thus, a roof structure was introduced to suppress ion bombardment, improving the etch selectivity from 14:1 to 23:1. The roof structure blocked ion bombardment caused by the plasma self-bias, which helped maintain a high etch selectivity by preventing ion-induced damage.

A comparison of O_2_ and N_2_ as additive gases revealed distinct effects. The addition of O_2_ to CF_4_ reduced the etch selectivity compared with pure CF_4_, whereas N_2_ significantly enhanced it, achieving values up to 35:1. This enhancement was attributed to the N_2_-induced promotion of CF_4_ dissociation, increased fluorine radical density, and N passivation on Si surfaces, which improved the SiGe-to-Si etch selectivity.

As the N_2_ flow rate increased, both the etch rate and selectivity initially increased and reached their maximum values. However, they began to decrease with further increases in N_2_ flow in both the blanket and ML structures. Optimal selectivity was achieved at moderate N_2_ flow rates.

XPS analysis confirmed the formation of Si–N bonds, contributing to the selective etching behavior. The increased fluorine radical density facilitated the formation of volatile Si(Ge)F_4_ by-products, which were subsequently pumped out of the chamber. After process optimization, the etch selectivity increased significantly—from 10:1 to 37:1. These findings demonstrate the importance of precisely tuning the gas chemistry and plasma parameters to achieve high selectivity and etch uniformity in Si/SiGe ML structures, offering valuable insights for next-generation 3D logic device fabrication.

## Figures and Tables

**Table 1:** Lateral etch depth of the SiGe layer in the SiGe/Si ML.

Thickness of the SiGe Layer	50 nm	30 nm	10 nm
CF_4_	183 nm	170 nm	145 nm
CF_4_ + O_2_	154 nm	100 nm	—
CF_4_ + N_2_	270 nm	242 nm	175 nm

**Table 2:** Summary of the etch rate and selectivity of Si_0.75_Ge_0.25_/Si ML under the following conditions: 20 sccm CF_4_, 10 sccm N_2_, and 30 mTorr for 60 s with the nonconducting plate.

Optimization		CF_4_ + N_2_ 30 mT
Etch rate (nm/min)	SiGe 50 nm	174
	SiGe 30 nm	143
	SiGe 10 nm	64
	Si	4.8
Selectivity (SiGe:Si)	SiGe 50 nm	37:1
	SiGe 30 nm	30:1
	SiGe 10 nm	14:1

**Table 3:** Peak fraction at different N_2_ flow rates.

**N_2_ Flow**	**Ge 3d**	**GeF_2_**
0 sccm	42.91	57.09
2 sccm	31.01	68.99
5 sccm	26.12	73.88
30 sccm	20.32	79.68

## Data Availability

The original contributions presented in this study are included in the article. Further inquiries can be directed to the corresponding authors.

## Abbreviations

The following abbreviations are used in this manuscript:

3D-DRAM	3D Dynamic random-access memory
CFET	Complementary field effect transistor
GAA	Gate-all-around
ICP-RIE	Inductively coupled plasma reactive ion etching
MFP	Mean free path
ML	Multilayer
sccm	Standard cubic centimeters per minute
SEM	Scanning electron microscopy
XPS	X-ray photoelectron spectroscopy
